# Development and External Validation of Mini PIERSdynamic Model for Predicting the Adverse Maternal Outcome of Preeclampsia in Low-Resource Settings

**DOI:** 10.1161/HYPERTENSIONAHA.126.27270

**Published:** 2026-09-18

**Authors:** Guiyou Yang, Tünde Montgomery-Csobán, Gerton Lunter, Wessel Ganzevoort, Sanne J. Gordijn, Paul Murray, Kimberley Kavanagh, Laura A. Magee, Henk Groen, Peter von Dadelszen

**Affiliations:** Department of Epidemiology (G.Y., G.L., H.G.), University Medical Center Groningen, University of Groningen, the Netherlands.; Department of Obstetrics and Gynecology (S.J.G.), University Medical Center Groningen, University of Groningen, the Netherlands.; Department of Cardiology, the First Affiliated Hospital of Shantou University Medical College, Guangdong, China (G.Y.).; Department of Mathematics and Statistics (T.M.-C., K.K.), University of Strathclyde, Glasgow, United Kingdom.; Department of Electronic and Electrical Engineering (P.M.), University of Strathclyde, Glasgow, United Kingdom.; Department of Obstetrics and Gynecology, Amsterdam University Medical Centers, the Netherlands (W.G.).; Amsterdam Reproduction and Development Research Institute, Amsterdam, the Netherlands (W.G.).; Institute of Women and Children’s Health, School of Life Course and Population Sciences, King’s College London, United Kingdom (L.A.M., P.v.D.).

**Keywords:** clinical decision-making, developing countries, forecasting, hypertension, pregnancy-induced, pre-eclampsia, pregnancy complications

## Abstract

**BACKGROUND:**

Preeclampsia remains a leading cause of maternal mortality, particularly in low-resource settings. We aimed to develop a dynamic prediction model accommodating repeated measurements for predicting adverse maternal outcomes in preeclampsia.

**METHODS:**

We utilized data from 7283 women with preeclampsia from the PIERS studies (Preeclampsia Integrated Estimate of Risk). Data from 6548 women were randomly split into a training set (80%) and an internal validation set (20%), while an independent cohort of 735 women was used for external validation. The outcome was a composite of maternal mortality and severe maternal morbidity. The mini PIERSdynamic model was fitted with the binary mixed model random forest algorithm. Threshold probabilities were calculated for 5 risk strata, ranging from very low to very high risk. Model performance was externally validated.

**RESULTS:**

In the development cohort, 785 (12.0%) women experienced an adverse maternal outcome, compared with 91 (12.4%) women in the external validation cohort. The final model retained all candidate predictors. Model performance was similar in internal and external validation. The daily area under the receiver-operating-characteristics curve remained around 0.75 on days 0 to 9 in internal validation and 0.70 in external validation, with peak values of 0.92 and 0.94, respectively. The area under the precision-recall curve declined over time as outcome incidence decreased. Observed daily event rates corresponded closely with the assigned risk strata.

**CONCLUSIONS:**

We developed the mini PIERSdynamic model and performed external validation, demonstrating its potential for dynamic prediction of adverse maternal outcomes in preeclampsia for up to 10 days after initial presentation. Further evaluation is required to verify its generalizability and clinical utility.

**Novelty and Relevance**

**What Is New?**

We developed and externally validated the mini PIERSdynamic model, a dynamic prediction model that updates the risk of adverse maternal outcomes of preeclampsia by incorporating temporal changes in maternal condition.

The model has easily accessible predictors, making it applicable especially in low-resource healthcare settings.

**What Is Relevant?**

Preeclampsia is a progressive disorder where maternal risk evolves over time. Consequently, risk assessed at admission may no longer accurately reflect a patient’s subsequent clinical status.

Dynamic prediction better reflects the changing clinical course of preeclampsia by incorporating longitudinal patient information, thereby enabling risk estimates to be updated as new observations become available.

A dynamic model based on readily accessible variables may facilitate broader implementation, particularly in settings where laboratory resources are limited.

**Clinical/Pathophysiological Implications?**

Dynamic risk prediction may support timely adjustment of care, such as appropriate referral of women whose clinical condition deteriorates.

The study reinforces the importance of viewing preeclampsia as a dynamic disease process rather than relying solely on a single baseline risk assessment.

Future studies should evaluate whether integrating dynamic prediction into routine clinical care improves maternal outcomes and resource utilization and whether incorporation of additional predictors can further enhance predictive performance.

Preeclampsia, defined as new-onset hypertension accompanied by significant end-organ dysfunction (most commonly affecting the liver and kidneys), with or without proteinuria, typically presents after 20 weeks of gestation.^1^ It is a major contributor to maternal and neonatal morbidity and mortality worldwide, affecting ≈2% to 4% of pregnancies.^2^ While the global maternal mortality ratio has declined by 34% from 339 deaths per 100 000 live births in 2000 to 223 deaths per 100 000 live births in 2020,^3^ preeclampsia continues to pose a substantial obstetric challenge that causes over 20 000 maternal deaths annually according to the latest report, along with 7 to 10× as many fetal or neonatal deaths.^4–9^

In the context of the global burden of preeclampsia, it is critical to examine the broader health inequalities that contribute to maternal mortality worldwide. Maternal mortality risks exhibit sharp regional disparities, with the highest lifetime risk observed in West and Central Africa, where 1 in 27 women faces maternal death, compared with 1 in 11 000 in Western Europe.^3^ In 2020, nearly every 2 minutes, a woman died from preventable pregnancy and childbirth-related causes, with 95% of these deaths occurring in low- and lower-middle-income countries.^10^ These disparities underscore the urgent need for tools tailored to resource-limited settings to achieve the Sustainable Development Goal of reducing maternal mortality to 70 deaths per 100 000 live births by 2030.^3^ Given that hypertensive disorders in pregnancy, including preeclampsia, contribute to ≈14% of maternal deaths,^3^ developing a prediction model for adverse outcomes of preeclampsia, specifically tailored for low-resource settings by relying solely on easily accessible predictors, is critically important.^11^ This approach ensures practicality and broad applicability where advanced diagnostic tools may not be readily available, while providing much-needed guidance to improve management of preeclampsia.

Although routine evaluations are conducted to track disease progression in clinical practice, existing models predicting adverse maternal outcomes of preeclampsia are typically trained using only measurements taken at admission, such as the Prediction of complications in Early-onset Preeclampsia (PREP) models,^12^ fullPIERS (Preeclampsia Integrated Estimate of Risk) model,^13^ PIERS-Machine Learning (ML) model,^14^ and miniPIERS model.^11^ Among them, only the miniPIERS model was designed specifically for low-resource settings. Laboratory parameters are known to fluctuate over time. Consequently, a single set of measurements provides only a snapshot of risk, which may be misleading and may fail to capture subsequent changes or overall trends in maternal condition. Incorporating longitudinal repeated measurements allows risk to be updated over time, aligning prediction with the dynamic nature of the disease and the clinical reality of ongoing monitoring. However, simply conducting serial prediction using static models that are trained with only baseline data has been shown to offer limited reliability.^15^

Therefore, in this study, we aimed to develop and validate a dynamic prediction model designed to incorporate repeated measurements as predictors for adverse maternal outcomes of preeclampsia in low-resource settings. The model focuses on using easily accessible parameters to enhance its practicality and is intended for use by healthcare professionals.

## Methods

### Data Availability

Because of the sensitive nature of the data collected for this study, requests to access the data set from qualified researchers trained in human subject confidentiality protocols may be sent to ma-piers@strath.ac.uk.

### Data Source

To optimize the sample size and improve the generalizability of the model, we obtained access to data from 7784 women with preeclampsia from the miniPIERS study (2008–2012),^11^ and fullPIERS development and external validation studies (year 2003–2016).^13,16^ These studies recruited women from various settings: the Americas, sub-Saharan Africa, South Asia, Europe, and Oceania. The definition of preeclampsia in these studies mirrors the 2021 International Society for the Study of Hypertension in Pregnancy criteria,^1^ capturing both the variable and multisystem presentation of the condition and reflecting the diversity of women encountered in routine clinical practice.^13^ The sites involved in this study typically managed women with preterm preeclampsia expectantly and, following the HYPITAT (Hypertension and Preeclampsia Intervention Trial At near Term) study,^17^ generally induced labor at term. The NHS Research Ethics Committee granted approval (REC reference: 02-03-033 on March 11, 2003) for this research project. Except for participants from the Canadian site, who were recruited through an audit, all other participants provided written informed consent. The study was performed in adherence to the principles outlined in the Declaration of Helsinki and reported according to the updated guidance for reporting clinical prediction models that use regression or machine learning methods (TRIPOD + AI statement [Transparent Reporting of a multivariable prediction model for Individual Prognosis Or Diagnosis + Artificial Intelligence statement]).^18^

### Inclusion and Exclusion Criteria

Details of the inclusion and exclusion criteria that resulted in the 7784 women have been described in the original studies.^11,13,16^ In short, women initially admitted with preeclampsia or who developed it after admission were included; women in spontaneous labor or those who had already developed adverse maternal outcome components before eligibility or data collection were excluded. Specifically, in this study, which was focused on incorporating repeated measurements for predicting adverse outcomes, we excluded women who lacked repeated measurements (ie, with only 1 row in the database). Moreover, we excluded women who developed adverse maternal outcomes within 48 hours of meeting eligibility criteria. We applied this exclusion because the miniPIERS model had been trained to predict adverse outcomes within this timeframe in low-resource settings.^11^ Our focus in this study was to develop a model that would be able to handle repeated measurements for predicting adverse outcomes without the influence of cases with rapid onset of adverse events.

### Model Development Data Set

The number of participants recruited in the miniPIERS study, the first stage of the fullPIERS study and its external validation studies was 7028. After screening the participants according to the inclusion and exclusion criteria, 6548 women were retained for model development. With a random split, data from 80% of the women (n=5240) were used for training the model and 20% (n=1308) for internal validation (Figure S1).

### External Validation Data Set

The fullPIERS study was a prospective multicenter study.^13^ Data collection in this study was conducted in 2 distinct stages. We considered the data (n=756) collected in the second stage to be suitable for external validation because of its sample size and vast geographic coverage. Based on the inclusion and exclusion criteria mentioned above, we excluded 21 women as they developed adverse maternal outcomes within 48 hours. Therefore, data from 735 women were retained for external validation (Figure S1).

### Adverse Maternal Outcome Components

The composite adverse maternal outcome was derived from a Delphi consensus.^13,19^ Because expectant management is recommended for women with preeclampsia until 37 weeks’ gestation unless clinical indications for delivery arise, and because most cases are late onset (≥34 weeks), we developed a model with a 2-week prediction window that captures ≈98% of adverse maternal outcomes.^20^ The adverse outcome components are consistent with other PIERS-based suite of models,^11,13,14^ defined as any of the following occurring within 48 hours after the initial evaluation for preeclampsia: (1) maternal mortality, (2) severe maternal morbidity involving major organ systems (eg, central nervous, cardiovascular, respiratory, renal, hepatic, hematologic), or (3) other serious complications (definition of individual components shown in Table S1). A 48-hour prediction horizon was selected because it represents a clinically meaningful timeframe for decisions regarding administration of corticosteroids for fetal lung maturation when indicated, intensified monitoring, and transfer to a tertiary center when appropriate.

### Predictors

As we intended to develop a model for low-resource settings, we selected easily accessible predictors (shown in Table S2) from the PIERS-ML model^14^ and miniPIERS model.^11^ Two more symptoms, that is, right upper quadrant or epigastric pain and nausea or vomiting, were considered as candidate predictors.^21^ While symptoms are generally suboptimal predictors due to the challenges in objective measurement, they are used to classify disease severity,^13,22^ which supports their potential in predicting adverse outcomes, particularly when laboratory parameters are not readily available.

### Missing Values

In the model development data set (n=6548), the percentage of missing values of the individual predictors ranged from 0.2% (gestational age at eligibility) to 57% (systolic/diastolic blood pressure), and the overall percentage of missing values was 12.3%. In the external validation data set (n=735), the percentage of missing values of the individual predictors ranged from 0.1% (gestational age at eligibility) to 51% (systolic/diastolic blood pressure), and the overall percentage of missing values was 8.8%. Since symptoms, for example, headache or visual disturbances, were patient-reported and documented in the medical record if they had ever occurred, missing values of symptom variables were considered missing not at random and filled using the last observation carried forward method.

Missing values for other variables were assumed to be either missing at random or completely at random. These values were imputed using the MICE R package,^23^ with 13 imputations performed for the model development data set and 9 imputations for the external validation data set, aligning with the recommendation that the number of imputations is at least equal to the percentage of incomplete cases.^24^

### Statistical Analysis

We used the Binary Mixed Model (bimm) random forest algorithm,^25^ as implemented in the R package bimm, for constructing the mini PIERSdynamic model. This algorithm first trains a random forest model, ignoring the correlation between repeated measurements from the same woman. The predicted probabilities from the random forest model are then used as covariates in a Bayesian generalized linear mixed-effects model, which accounts for within-subject correlation.^25^ To test the performance of the candidate models and select the best model, we quantified and compared the daily area under the receiver-operating-characteristics curve (AUC-ROC) and area under the precision-recall curve (AUC-PRC) from day 0 (date of eligibility) to day 14 (14 days following eligibility). The final mini PIERSdynamic model was aimed to stratify women with preeclampsia into different risk strata. Aligning with the PIERS-ML model, we used the same values of likelihood ratios to determine different risk strata: very low risk (negative likelihood ratio <0.10), low risk (negative likelihood ratio between 0.10 and 0.20), high risk (positive likelihood ratio between 5.0 and 10.0), very high risk (positive likelihood ratio >10.0), and moderate risk outside these ranges.^14^ As the sample size in the external validation data set was relatively low, we utilized data from day 0 in the internal validation data set (n=1308) for calculating the cutoff values of the predicted risk that meet the preset likelihood ratios mentioned above. Subsequently, these cutoff values were applied to the external validation data set to calculate the daily number of women and the incidence rate of adverse maternal outcomes within each risk stratum.

We performed all analyses in RStudio (version 2024.04.01+748, R, version 4.3.3). Other R packages used in this study include haven (version 2.5.4),^26^ skimr (version 2.1.5),^27^ tidyverse (version 2.0.0),^28^ magrittr (version 2.0.3),^29^ bimm (version 0.1.0),^25^ epiR (version 2.0.78),^30^ pROC (version 1.18.5),^31^ tictoc (version 1.2.1),^32^ reshape2 (version 1.4.4),^33^ and patchwork (version 1.2.0).^34^

## Results

The baseline characteristics of participants are presented in Table 1. Women were categorized into 4 subgroups based on maternal outcomes and the purpose of their data: model development or external validation. Among the 6548 women in the model development data set, 785 (12.0%) had the defined adverse maternal outcome; a similar incidence rate (91/735, 12.4%) was observed in the external validation data set. The median maternal age at the expected delivery date in each subgroup was 30 to 32 years. In the model development data set, the proportions of White, Asian, and Black women were similar among those without adverse maternal outcomes (30.3%, 30.0%, and 27.1%, respectively) and those with adverse maternal outcomes (27.5%, 33.5%, and 28.2%, respectively). In the external validation data set, the proportions of White, Asian, and Black women were highly unbalanced both among those without adverse maternal outcomes (4.8%, 19.4%, and 63.4%, respectively) and those with adverse maternal outcomes (3.3%, 18.7%, and 71.4%, respectively). In addition, the median (interquartile range) of the national maternal mortality rates (per 100 000 live births) in the model development data set was 12 (11–194) for the group without the defined adverse outcome and 12 (11–217) for the group with the defined adverse outcome, while that in the external validation data set was 13 (10–13) for both groups.

**Table 1.**
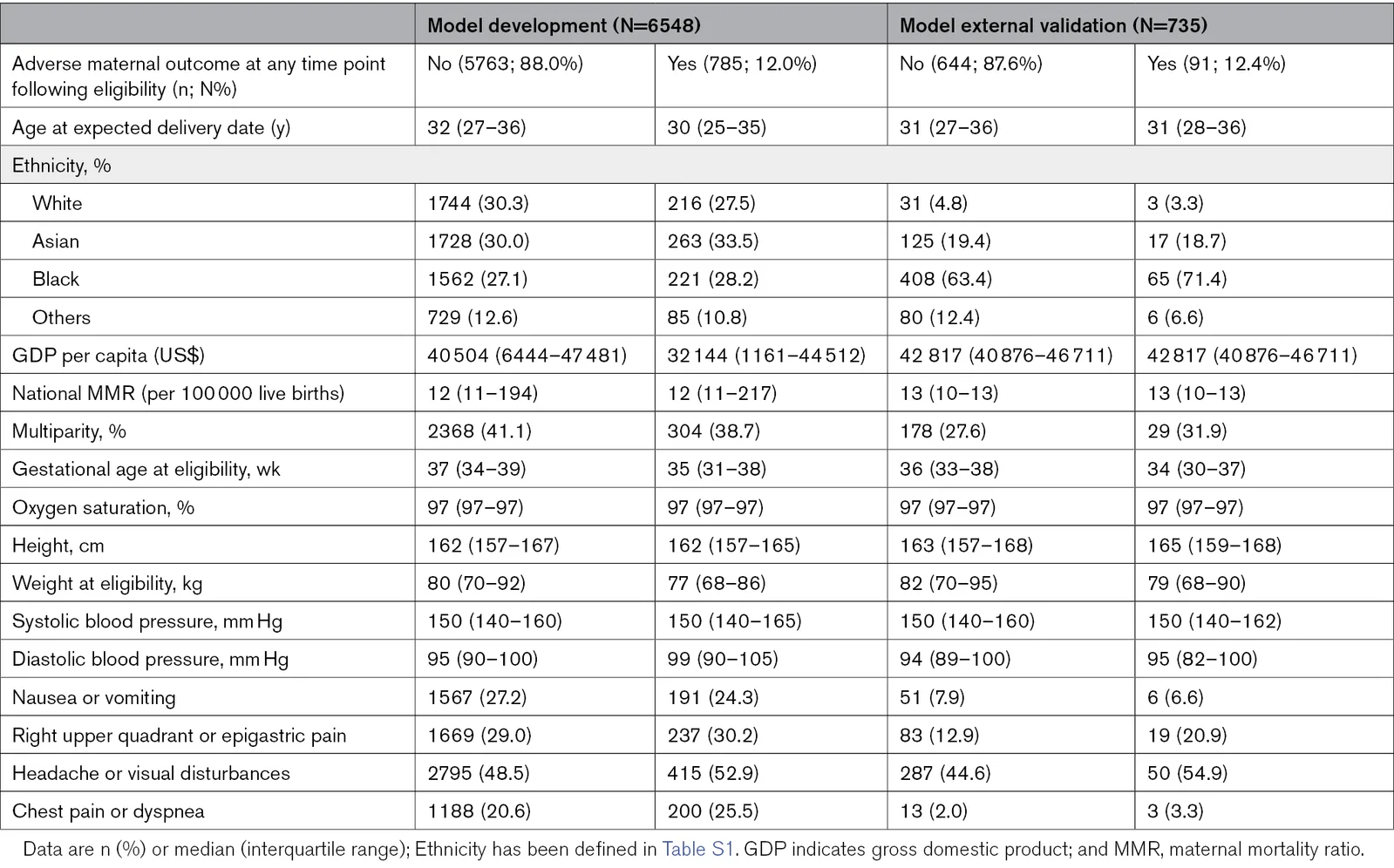
Baseline Characteristics of Participants With and Without Maternal Adverse Outcome

The final mini PIERSdynamic model included all the predictors and candidate predictors from Table S2. Figure 1 shows the discriminative capacity of the model from day 0 to day 14 in the internal validation data set. During the 2-week period, the daily number of women providing data decreased from over 1000 to <500. Most of the daily AUC-ROC values were close to 0.75, with the highest AUC-ROC being 0.92 on day 9. As the number of adverse maternal events declined, the 95% CI of the AUC-ROC value widened substantially from day 10 onward. After day 9, the lower bound of the 95% CIs approached or fell below 0.5. The results in the external validation data set are shown in Figure 2. The daily number of women decreased from over 600 to <200 from day 0 to day 14. The daily AUC-ROC values fluctuated around 0.7 and peaked at 0.94 on day 10. Most of the lower bounds of the 95% CIs of the AUC-ROC values fell below 0.5 after day 3.

**Figure 1.**
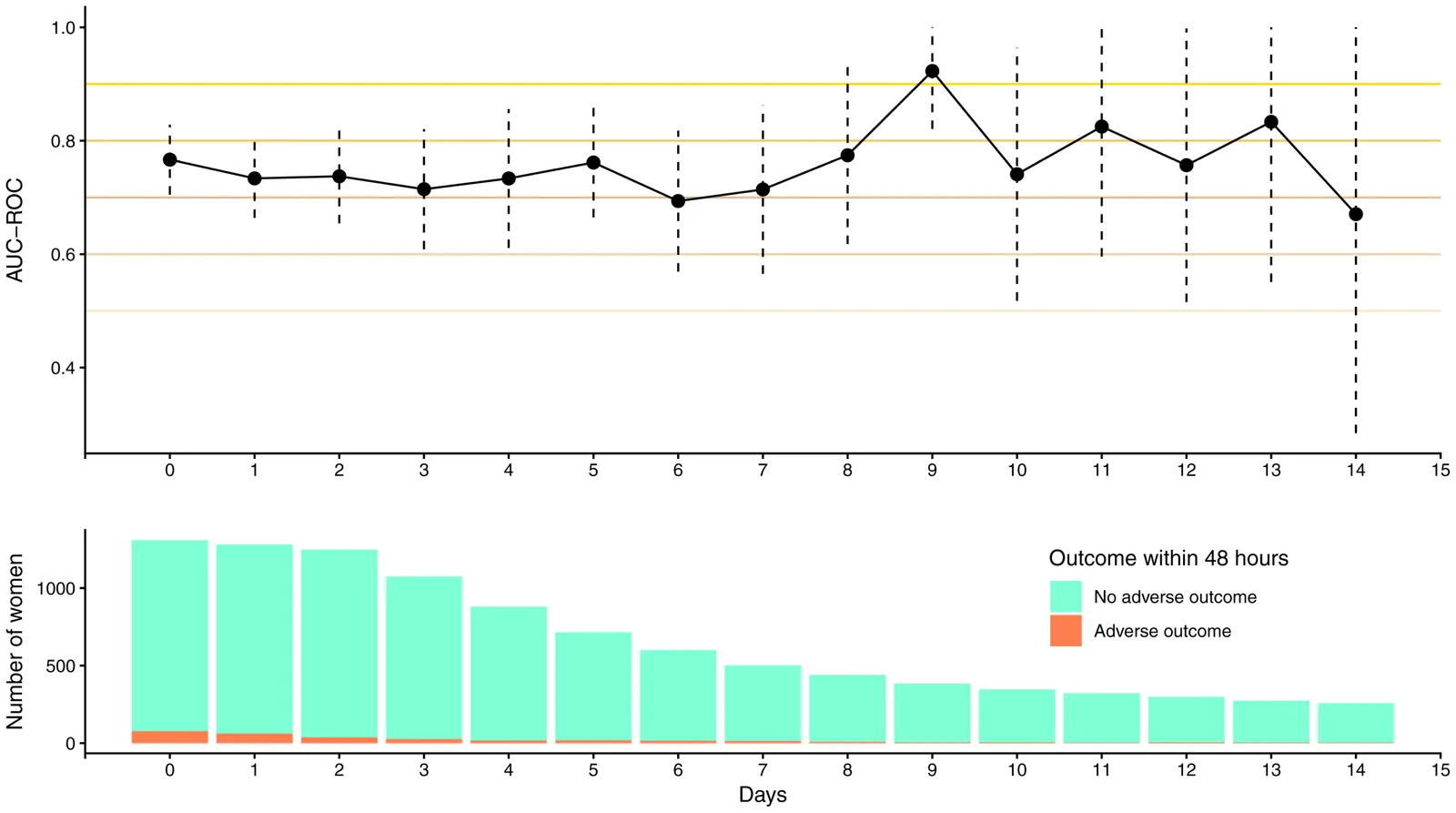
Daily area under the receiver operating characteristic curve (AUC-ROC) for predicting adverse maternal outcome occurring within 48 hours and daily outcome incidence (internal validation).

**Figure 2.**
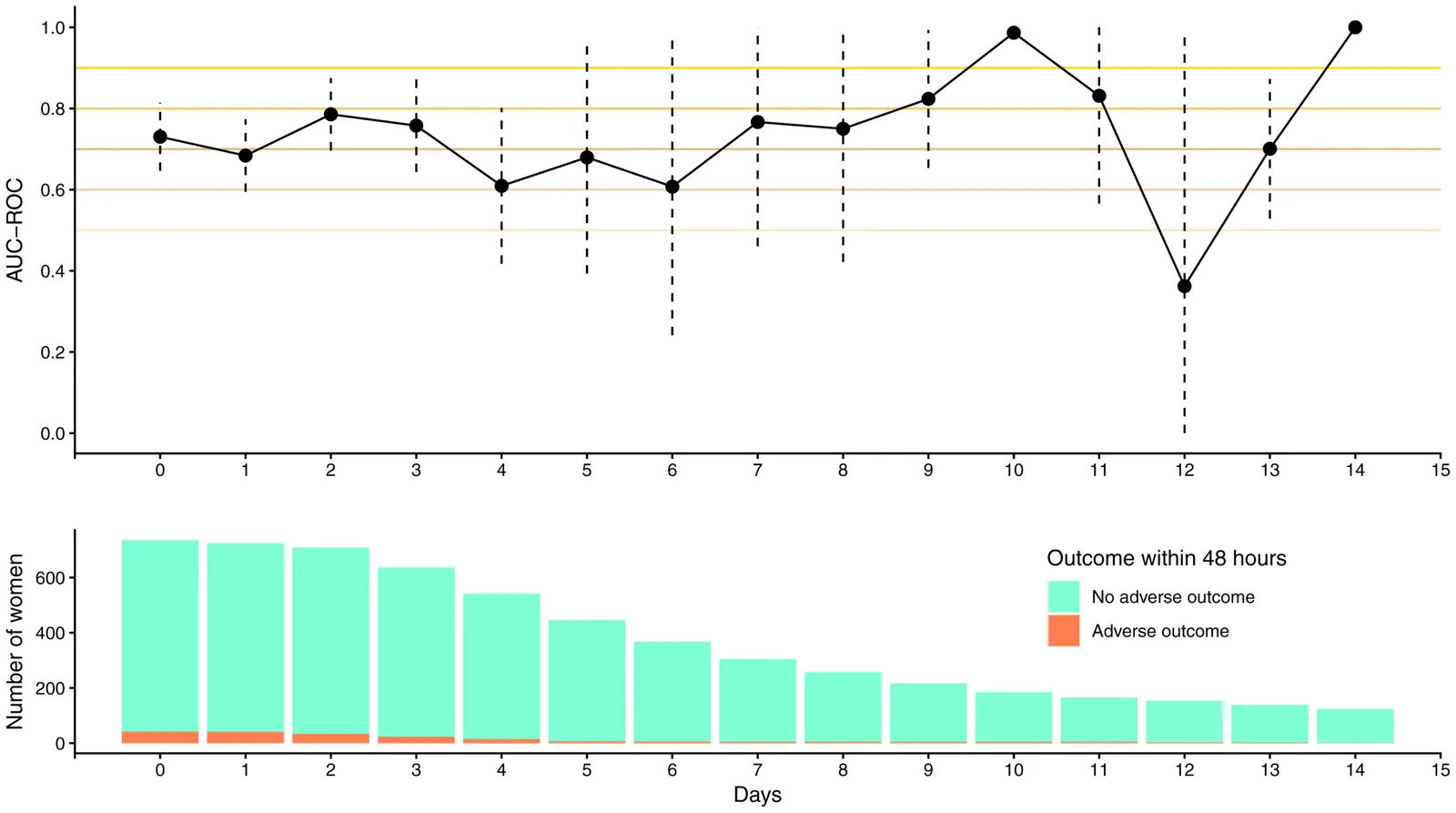
Daily area under the receiver operating characteristic curve (AUC-ROC) for predicting adverse maternal outcome occurring within 48 hours and daily outcome incidence (external validation).

Figure 3 illustrates the daily AUC-PRC values of the mini PIERSdynamic model in the internal validation data set. The AUC-PRC value was highest on day 0 (0.31) and lowest on day 10 (0.04). There was a general trend of decline, with the lower bound of the AUC-PRC value approaching the daily fraction of adverse maternal outcomes within 48 hours by day 3, except for the AUC-PRC value on day 9 (0.21). The AUC-PRC values in the external validation data set are depicted in Figure 4. Generally, the AUC-PRC values showed a tendency to decrease from day 0 (0.27) to day 9 (0.08) and converge toward the daily fraction of predicted adverse maternal outcomes. However, the highest AUC-PRC value was observed on day 10 (0.42), when there was also a slight increase in the fraction of adverse outcomes.

**Figure 3.**
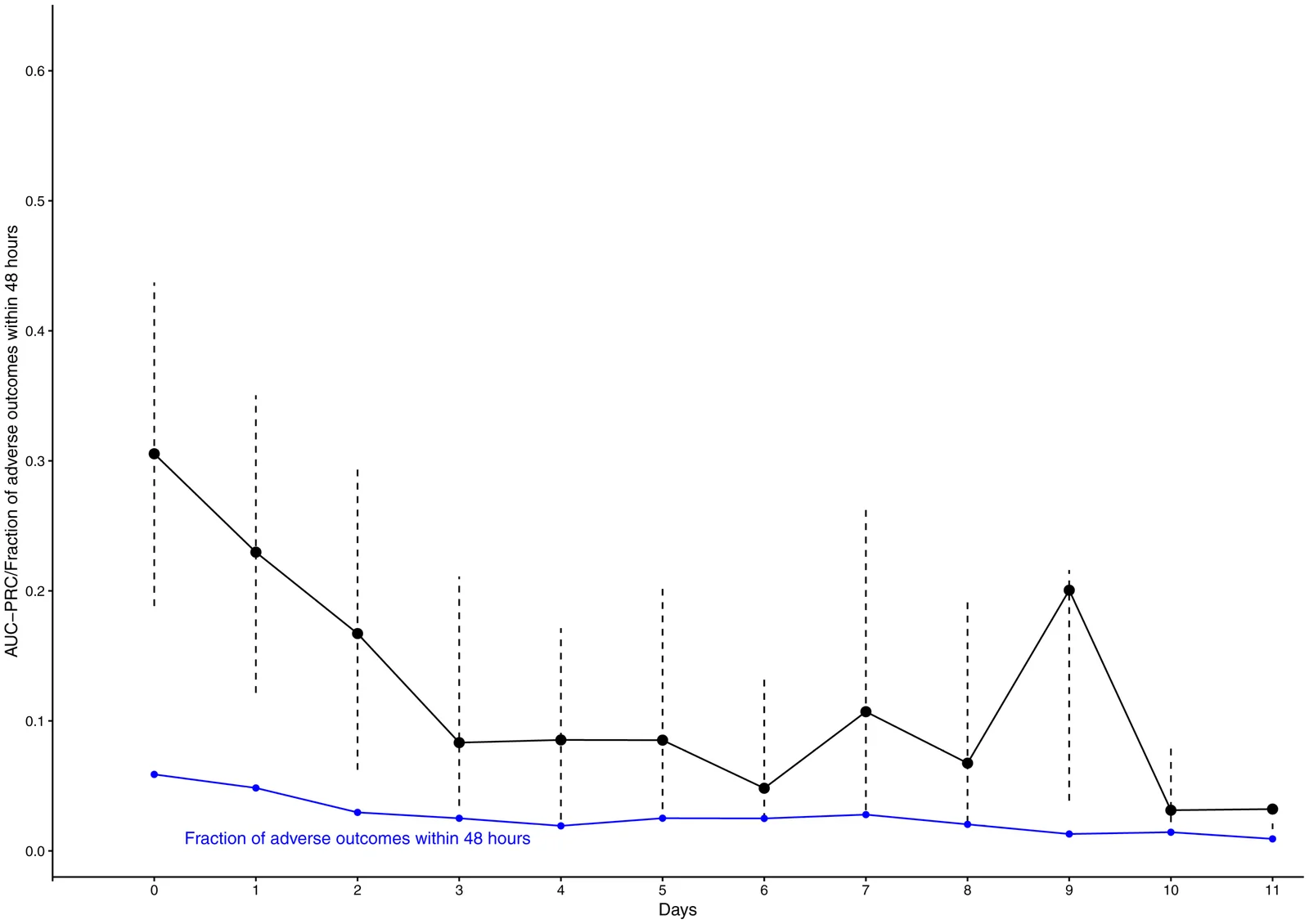
Daily area under the precision-recall curve (AUC-PRC) for predicting adverse maternal outcome occurring within 48 hours (internal validation).

**Figure 4.**
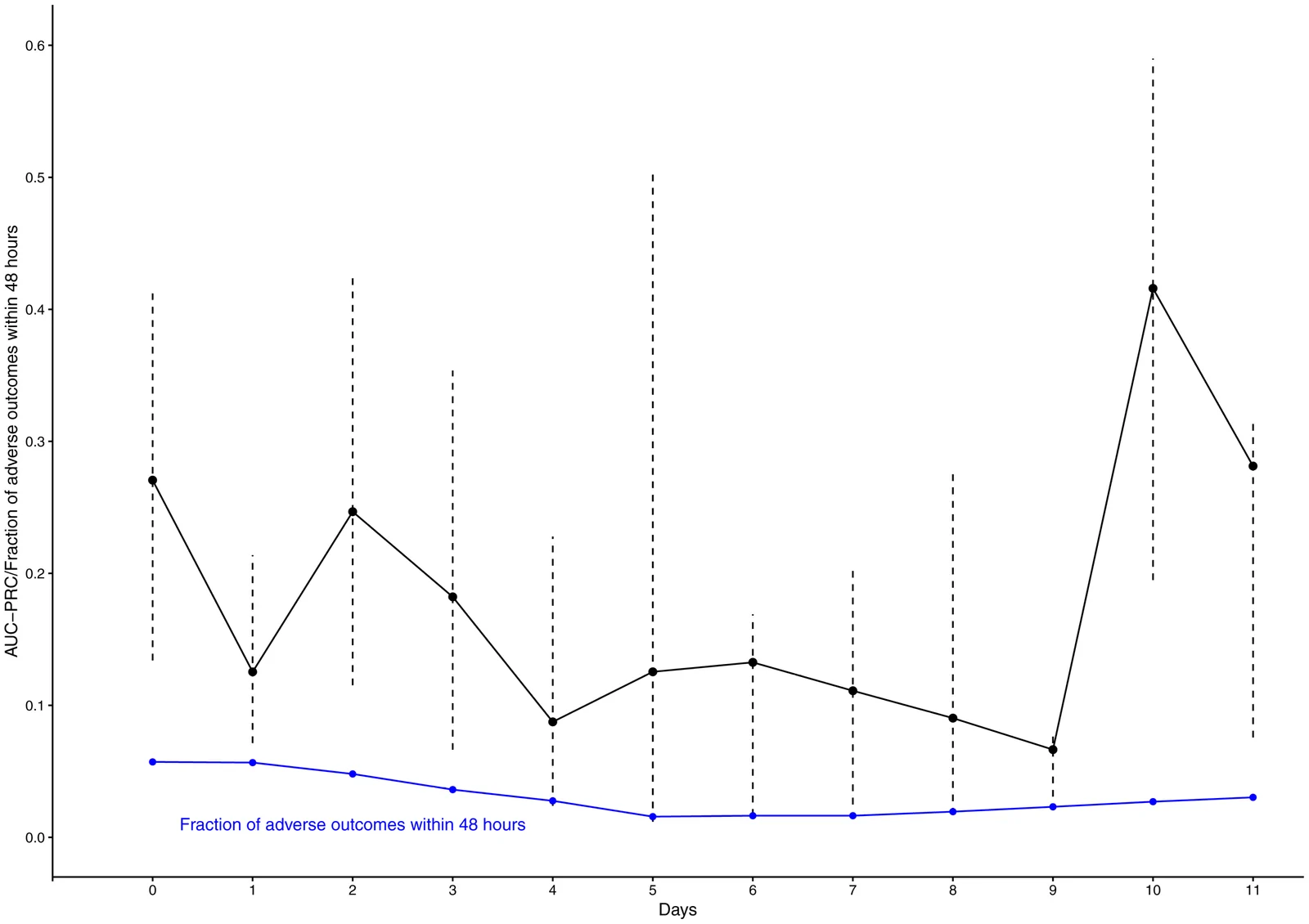
Daily area under the precision-recall curve (AUC-PRC) for predicting adverse maternal outcome occurring within 48 hours (external validation).

We calculated the paired threshold probabilities and likelihood ratios using the internal validation data set. Membership in the risk strata from very low risk (negative likelihood ratio, LR− <0.1), low risk (LR− ≥0.1 and LR− ≤0.2), moderate risk (LR− >0.2 and positive LR+ <5), high risk (LR+ ≥5 and LR+ ≤10) to very high risk (LR+ >10) corresponds to the following predicted probability ranges: <0.002, 0.002 to 0.007, >0.007 to <0.10, 0.10 to 0.24, and > 0.24, respectively.

The results of applying the threshold probabilities for the 5 risk strata to the external validation data set are depicted in Table 2. From day 0 to day 10, most women were predicted to be at moderate risk of the adverse maternal outcome, for example, 587 of 735 (80%) women on day 0, with the daily incidence rate in this group ranging from 0.00 to 0.04. At most 1 woman was predicted to be at very low risk on a daily basis, with no adverse maternal outcomes observed in this group. There were 0 to 12 women each day in the low-risk group, with no adverse maternal outcome recorded except on day 4 (2/6, incidence: 33%). The number of women in the high-risk group decreased from 123 on day 0 to 12 on day 10, with the incidence rate ranging from 0% to 42%. In the very high-risk group, the number of women was between 0 and 17, with most incidence rates above 40%.

**Table 2.**
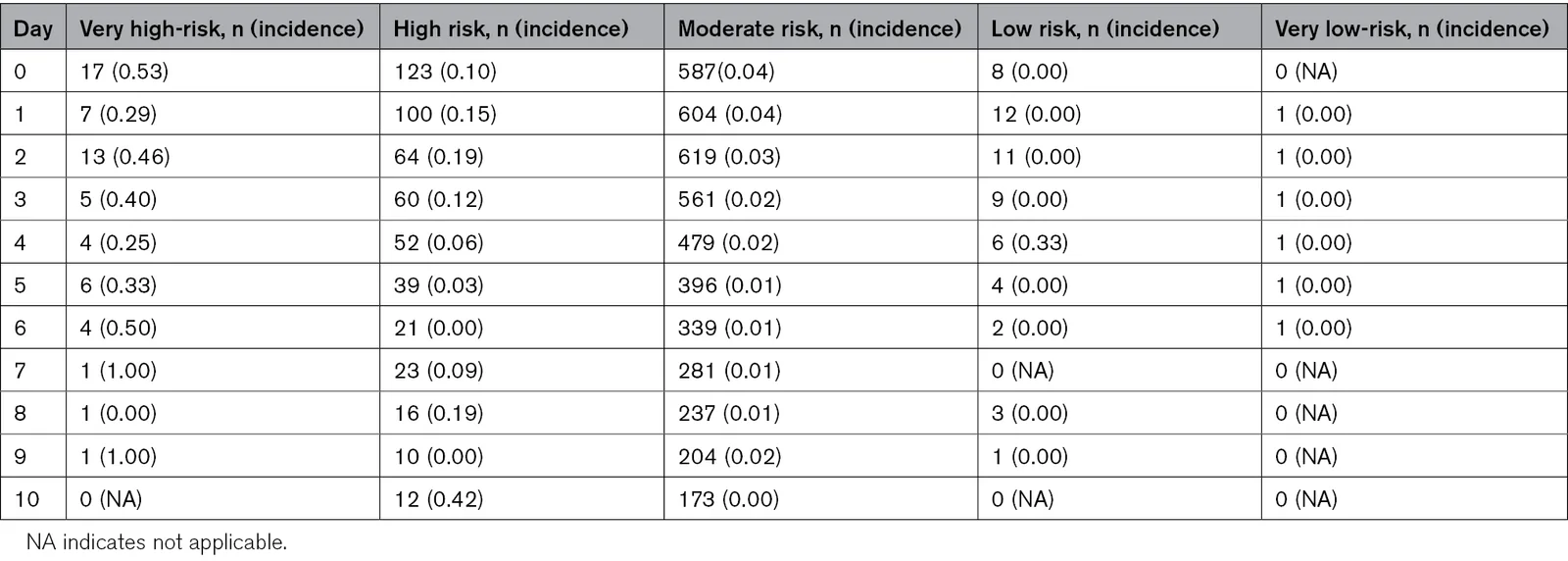
Daily Number of Women and Incidence Rate in Each Risk Stratum

## Discussion

### Interpretation of the Main Results

Using data from 7283 women, we developed and externally validated the mini PIERSdynamic model targeted at low-resource settings, permitting serial prediction of the adverse maternal outcomes within the following 48 hours, and complementing the more replete PIERSdynamic model that is most applicable to well-resourced settings (T. Montgomery-Csobán, unpublished data, 2026). The results of internal validation demonstrated a marked decline in model performance from day 10 onward. The performance in external validation closely aligned with internal validation, suggesting a low risk of model overfitting despite the notable sociodemographic differences between the development and external validation data sets. However, the lower bound of the 95% CI of the AUC-ROC values approached or fell below 0.5 in earlier days in external validation, presumably attributable to the relatively small sample size. The results of the data-driven multiple risk strata analysis demonstrated a trend of progressive increase in the risk of adverse maternal outcomes with higher risk stratum, indicating good calibration capacity of the model.

Both the AUC-ROC and the AUC-PRC values indicated that model performance had a peak on day 9 during internal validation and on day 10 during external validation. Although this pattern might suggest improved discrimination at later prediction time points, these estimates should be interpreted cautiously because the number of women and outcome events decreased substantially over time, resulting in greater statistical uncertainty. Consequently, the observed peak in performance is more likely to reflect imprecision than a true improvement in model performance. While repeated measurements may provide additional prognostic information, their incremental value over a baseline-only model remains uncertain and may be difficult to estimate reliably at later time points because of the limited number of women and outcome events.

To our knowledge, the mini PIERSdynamic model is the first dynamic prediction model intended for use in low-resource settings, accommodating repeated measurements of predictors for prediction. Compared with the predictors of the static miniPIERS model,^11^ we removed dipstick proteinuria from the dynamic model owing to its low sensitivity and specificity.^35^ In contrast, to accommodate the multinational and multisetting nature of our data sets, we incorporated several predictors derived from the PIERS-ML model,^14^ such as proxy variables for the quality of healthcare, namely gross domestic product per capita and maternal mortality ratio, which are considered important factors as they do not necessarily correlate with each other. For instance, while the gross domestic product per capita of Cyprus is ≈9× that of Sri Lanka, the maternal mortality ratio in Cyprus is typically twice that of Sri Lanka in recent years.^36^ In addition, nausea/vomiting beyond mid-pregnancy and ethnicity, which were not retained as predictors in the miniPIERS model, increased the predictive accuracy of the mini PIERSdynamic model. This improvement may be attributable to the different modeling approaches used: the miniPIERS model relied on logistic regression, whereas the mini PIERSdynamic model is based on machine learning. Machine learning algorithms are known for their ability to capture complex relationships among predictors,^37^ potentially revealing the benefits of incorporating these additional variables.

### Strengths

There are several strengths in this modeling study. First, although this model was designed for use in low-resource settings, we used data from various settings for training this model to enhance its robustness and adaptability to varying socioeconomic and demographic conditions in those countries. Second, we incorporated repeated measurements, such as blood pressure values, for prediction, so that the dynamic clinical information of each individual woman could be leveraged for tailored decision-making. Finally, we selected only readily accessible predictors for the model yet achieved good performance.

### Limitations

Some limitations should be noted for this study. As this model was targeted for use in low-resource settings, this machine learning-based model might seem complicated. However, it does not require more resources than most traditional prediction models. A simple calculator could be easily implemented in clinical practice, allowing clinicians to enter the relevant data through a point-and-click interface and obtain an updated risk estimate. Nevertheless, successful implementation will depend not only on the accessibility of the prediction tool but also on the accuracy of the data entered. Appropriate and focused education of both healthcare providers and patients may therefore be important to support reliable data collection and model use in clinical practice. Future implementation studies should evaluate these aspects alongside the clinical benefit of using the model.

Moreover, given the substantially larger volume of data available from high-income settings compared with low-resource settings, and considering the intended application of the model, we used all data from low-resource settings for model training and externally validated the model with data from high-income settings. Future research should externally validate this model using data from low-resource settings to further verify its generalizability and applicability.

Another limitation is that most women were classified as moderate risk, which is the least informative risk stratum.^11^ While this distribution is understandable and reflects clinical reality, future research aimed at updating the model would benefit from employing larger data sets with external validation incorporating data from low-resource settings to improve its discrimination capacity.

Finally, the prediction window may currently be relatively short for women with early onset preeclampsia. Future research should investigate whether the dynamic prediction framework can be extended to a longer prediction horizon, especially for early onset preeclampsia, while maintaining adequate predictive performance. Such models could further support individualized surveillance schedules and care planning, particularly in resource-limited settings.

### Conclusions

In this study, we developed the mini PIERSdynamic model and performed external validation, demonstrating its potential to provide dynamic risk prediction of adverse maternal outcomes of preeclampsia for up to 10 days following first admission. Given the comparable performance of the model in internal and external validation, we speculate that the model might have a low risk of overfitting. However, given the limited sample size and number of events in the validation cohort, future research is warranted to further verify and improve the performance and prediction window of this model.

### Perspectives

Dynamic risk prediction has the potential to shift the management of preeclampsia from a single time-point assessment to continuous evaluation of maternal risk as the clinical condition evolves. Such an approach may improve clinical decision-making across diverse healthcare settings by enabling risk assessment to be updated as new clinical information becomes available. The use of routinely collected and easily accessible clinical predictors further enhances the applicability of the mini PIERSdynamic model, particularly in low-resource settings where advanced laboratory tests and specialized diagnostic facilities may not be consistently available.

Beyond its potential clinical application, this study highlights the importance of developing prediction models that explicitly account for the longitudinal nature of disease progression. Future studies should evaluate the clinical impact of integrating dynamic risk prediction into routine obstetric care, including whether its use improves maternal outcomes, referral decisions, and healthcare efficiency. Additional external validation in geographically diverse populations and healthcare systems is needed to establish the model’s generalizability. Furthermore, incorporating emerging predictors, such as point-of-care biomarkers or ultrasound findings where available, may further improve predictive performance while maintaining applicability in low-resource settings. Ultimately, robust dynamic prediction tools could contribute to more personalized management of preeclampsia and support global efforts to reduce preventable maternal morbidity and mortality.

## Article Information

### Acknowledgments

The authors extend their heartfelt gratitude to all individuals who made contributions to the PIERS (Preeclampsia Integrated Estimate of Risk) data set, as well as to the collaborating coinvestigators whose partnership and dedication greatly supported this work.

### Author Contributions

All authors conceptualized this project together. P. von Dadelszen, L.A. Magee, and H. Groen obtained the data. G. Yang and T. Montgomery-Csobán cleaned the data and performed data analyses. G. Yang drafted the manuscript; all authors contributed substantially to revisions and approved the submitted version.

### Disclosures

None.

## Supplementary Material



## References

[1] Magee LA Brown MA Hall DR Gupte S Hennessy A Karumanchi SA Kenny LC McCarthy F Myers J Poon LC. The 2021 International Society for the Study of Hypertension in Pregnancy classification, diagnosis & management recommendations for international practice. Pregnancy Hypertens. 2022;27:148–169. doi: 10.1016/j.preghy.2021.09.008 10.1016/j.preghy.2021.09.008 35066406

[2] Magee LA Nicolaides KH von Dadelszen P. Preeclampsia. N Engl J Med. 2022;386:1817–1832. doi: 10.1056/NEJMra2109523 10.1056/NEJMra2109523 35544388

[3] UNICEF. Maternal mortality declined by 34 per cent between 2000 and 2020. Accessed November 27, 2024. https://data.unicef.org/topic/maternal-health/maternal-mortality/

[4] GBD 2015 Child Mortality Collaborators. Global, regional, national, and selected subnational levels of stillbirths, neonatal, infant, and under-5 mortality, 1980–2015: a systematic analysis for the Global Burden of Disease Study 2015. Lancet. 2016;388:1725–1774. doi: 10.1016/S0140-6736(16)31575-6 10.1016/S0140-6736(16)31575-6 PMC5224696 27733285

[5] GBD 2015 Maternal Mortality Collaborators. Global, regional, and national levels of maternal mortality, 1990–2015: a systematic analysis for the Global Burden of Disease Study 2015. Lancet. 2016;388:1775–1812. doi: 10.1016/S0140-6736(16)31470-2 10.1016/S0140-6736(16)31470-2 PMC5224694 27733286

[6] GBD 2019 Diseases and Injuries Collaborators. Global burden of 369 diseases and injuries in 204 countries and territories, 1990–2019: a systematic analysis for the Global Burden of Disease Study 2019. Lancet. 2020;396:1204–1222. doi: 10.1016/S0140-6736(20)30925-9 10.1016/S0140-6736(20)30925-9 PMC7567026 33069326

[7] Duley L. The global impact of pre-eclampsia and eclampsia. Semin Perinatol. 2009;33:130–137. doi: 10.1053/j.semperi.2009.02.010 10.1053/j.semperi.2009.02.010 19464502

[8] Duley L Meher S Abalos E. Management of pre-eclampsia. BMJ. 2006;332:463–468. doi: 10.1136/bmj.332.7539.463 10.1136/bmj.332.7539.463 PMC1382544 16497761

[9] Wu P Green M Myers JE. Hypertensive disorders of pregnancy. BMJ. 2023;381:e071653. doi: 10.1136/bmj-2022-071653 10.1136/bmj-2022-071653 37391211

[10] WHO. Maternal mortality. Accessed November 27, 2024. https://www.who.int/news-room/fact-sheets/detail/maternal-mortality

[11] Payne BA Hutcheon JA Ansermino JM Hall DR Bhutta ZA Bhutta SZ Biryabarema C Grobman WA Groen H Haniff F; miniPIERS Study Working Group. A risk prediction model for the assessment and triage of women with hypertensive disorders of pregnancy in low-resourced settings: the miniPIERS (Pre-eclampsia Integrated Estimate of Risk) multi-country prospective cohort study. PLoS Med. 2014;11:e1001589. doi: 10.1371/journal.pmed.1001589 10.1371/journal.pmed.1001589 PMC3897359 24465185

[12] Thangaratinam S Allotey J Marlin N Dodds J Cheong-See F von Dadelszen P Ganzevoort W Akkermans J Kerry S Mol BW; PREP Collaborative Network. Prediction of complications in early-onset pre-eclampsia (PREP): development and external multinational validation of prognostic models. BMC Med. 2017;15:68. doi: 10.1186/s12916-017-0827-3 10.1186/s12916-017-0827-3 PMC5372261 28356148

[13] von Dadelszen P Payne B Li J Ansermino JM Broughton Pipkin F Cote AM Douglas MJ Gruslin A Hutcheon JA Joseph KS; PIERS Study Group. Prediction of adverse maternal outcomes in pre-eclampsia: development and validation of the fullPIERS model. Lancet. 2011;377:219–227. doi: 10.1016/S0140-6736(10)61351-7 10.1016/S0140-6736(10)61351-7 21185591

[14] Montgomery-Csoban T Kavanagh K Murray P Robertson C Barry SJE Vivian Ukah U Payne BA Nicolaides KH Syngelaki A Ionescu O; PIERS Consortium. Machine learning-enabled maternal risk assessment for women with pre-eclampsia (the PIERS-ML model): a modelling study. Lancet Digit Health. 2024;6:e238–e250. doi: 10.1016/S2589-7500(23)00267-4 10.1016/S2589-7500(23)00267-4 PMC10983826 38519152

[15] Yang G Montgomery-Csoban T Ganzevoort W Gordijn SJ Kavanagh K Murray P Magee LA Groen H von Dadelszen P. Consecutive prediction of adverse maternal outcomes of preeclampsia, using the PIERS-ML and fullPIERS models: a multicountry prospective observational study. PLoS Med. 2025;22:e1004509. doi: 10.1371/journal.pmed.1004509 10.1371/journal.pmed.1004509 PMC11793762 39903757

[16] Ukah UV Payne B Karjalainen H Kortelainen E Seed PT Conti-Ramsden FI Cao V Laivuori H Hutcheon J Chappell L; fullPIERS Group. Temporal and external validation of the fullPIERS model for the prediction of adverse maternal outcomes in women with pre-eclampsia. Pregnancy Hypertens. 2019;15:42–50. doi: 10.1016/j.preghy.2018.01.004 10.1016/j.preghy.2018.01.004 30825926

[17] Koopmans CM Bijlenga D Groen H Vijgen SM Aarnoudse JG Bekedam DJ van den Berg PP de Boer K Burggraaff JM Bloemenkamp KW; HYPITAT study group. Induction of labour versus expectant monitoring for gestational hypertension or mild pre-eclampsia after 36 weeks’ gestation (HYPITAT): a multicentre, open-label randomised controlled trial. Lancet. 2009;374:979–988. doi: 10.1016/S0140-6736(09)60736-4 10.1016/S0140-6736(09)60736-4 19656558

[18] Collins GS Moons KGM Dhiman P Riley RD Beam AL Van Calster B Ghassemi M Liu X Reitsma JB van Smeden M. TRIPOD+AI statement: updated guidance for reporting clinical prediction models that use regression or machine learning methods. BMJ. 2024;385:e078378. doi: 10.1136/bmj-2023-078378 10.1136/bmj-2023-078378 PMC11019967 38626948

[19] Milholland AV Wheeler SG Heieck JJ. Medical assessment by a Delphi group opinion technic. N Engl J Med. 1973;288:1272–1275. doi: 10.1056/NEJM197306142882405 10.1056/NEJM197306142882405 4703316

[20] National Institute for Health and Care Excellence. Hypertension in pregnancy: diagnosis and management. 2023. 31498578

[21] Preeclampsia Foundation. SIGNS & SYMPTOMS. Accessed December 16, 2024. https://www.preeclampsia.org/signs-and-symptoms

[22] Brown MA Hague WM Higgins J Lowe S McCowan L Oats J Peek MJ Rowan JA Walters BN; Austalasian Society of the Study of Hypertension in Pregnancy. The detection, investigation and management of hypertension in pregnancy: full consensus statement. Aust N Z J Obstet Gynaecol. 2000;40:139–155. doi: 10.1111/j.1479-828x.2000.tb01137.x 10.1111/j.1479-828x.2000.tb01137.x 10925900

[23] van Buuren S Groothuis-Oudshoorn K. mice: multivariate imputation by chained equations in R. J Stat Softw. 2011;45:1–67. doi: 10.32614/cran.package.mice

[24] White IR Royston P Wood AM. Multiple imputation using chained equations: issues and guidance for practice. Stat Med. 2011;30:377–399. doi: 10.1002/sim.4067 10.1002/sim.4067 21225900

[25] Speiser JL Wolf BJ Chung DJ Karvellas CJ Koch DG Durkalski VL. BiMM forest: a random forest method for modeling clustered and longitudinal binary outcomes. Chemometrics and intelligent laboratory systems : an international journal sponsored by the Chemometrics Society. 2019;185:122–134. doi: 10.1016/j.chemolab.2019.01.002 10.1016/j.chemolab.2019.01.002 PMC6813794 31656362

[26] Wickham H Miller E Smith D. haven: Import and Export “SPSS,” “Stata” and “SAS” Files. Accessed May 5, 2023. https://cran.r-project.org/web/packages/haven/index.html

[27] Waring E Quinn M McNamara A Arino de la Rubia E Zhu H Ellis S. skimr: Compact and Flexible Summaries of Data. Accessed May 5, 2023. https://cran.r-project.org/web/packages/skimr/index.html

[28] Wickham H. tidyverse: Easily Install and Load the “Tidyverse”. Accessed May 5, 2023. https://cran.r-project.org/web/packages/tidyverse/index.html

[29] Bache SM Wickham H. magrittr: A Forward-Pipe Operator for R. Accessed May 5, 2023. https://cran.r-project.org/web/packages/magrittr/index.html

[30] Stevenson M Sergeant E. epiR: Tools for the Analysis of Epidemiological Data. Accessed May 5, 2023. https://cran.r-project.org/web/packages/epiR/index.html

[31] Robin X Turck N Hainard A Tiberti N Lisacek F Sanchez JC Müller M. pROC: Display and Analyze ROC Curves. Accessed May 5, 2023. https://cran.r-project.org/web/packages/pROC/index.html

[32] Izrailev S. tictoc: Functions for Timing R Scripts, as Well as Implementations of “Stack” and “StackList” Structures. Accessed May 5, 2023. https://cran.r-project.org/web/packages/tictoc/index.html

[33] Wickham H. reshape2: Flexibly Reshape Data: A Reboot of the Reshape Package. Accessed May 5, 2023. https://cran.r-project.org/web/packages/reshape2/index.html

[34] Pedersen TL. patchwork: The Composer of Plots. Accessed May 5, 2023. https://cran.r-project.org/web/packages/patchwork/index.html

[35] Jones C Jakubowski BE Stevens R Roberts N McManus RJ Tucker KL. The diagnostic accuracy of visual versus automated dipstick proteinuria testing in pregnancy: a systematic review and meta-analysis. Pregnancy Hypertens. 2024;35:73–81. doi: 10.1016/j.preghy.2024.01.133 10.1016/j.preghy.2024.01.133 38262144

[36] WHO. Maternal mortality ratio (per 100 000 live births). Accessed January 3, 2025. https://data.who.int/indicators/i/C071DCB/AC597B1

[37] Sarker IH. Machine learning: algorithms, real-world applications and research directions. SN Comput Sci. 2021;2:160. doi: 10.1007/s42979-021-00592-x 10.1007/s42979-021-00592-x PMC7983091 33778771

