# Supplementary material for: Development and External Validation of Mini PIERSdynamic Model for Predicting the Adverse Maternal Outcome of Preeclampsia in Low-Resource Settings
Source: Hypertension. 2026 Sep 18;83(11):e27270. doi: 10.1161/HYPERTENSIONAHA.126.27270 (PMC13680779; doi:10.1161/HYPERTENSIONAHA.126.27270)
Supplement: Supplementary file 2 [file hyp-83-e27270-s001.docx]

**Development and external validation of mini PIERSdynamic model for predicting the adverse maternal outcome of pre-eclampsia in low-resource settings**

**Short title**: a dynamic prediction model for pre-eclampsia outcome

Guiyou Yang^1,2$^, Tünde Montgomery-Csobán^3$^, Gerton Lunter^1^**,** Wessel Ganzevoort^4,5^, Sanne J Gordijn^6^, Paul Murray^7^, Kimberley Kavanagh^3^, Laura A. Magee^8^, Henk Groen^1*^, Peter von Dadelszen^8*^

**Affiliations**

1. Department of Epidemiology, University Medical Center Groningen, University of Groningen, Groningen, The Netherlands

2. Department of Cardiology, the First Affiliated Hospital of Shantou University Medical College, Shantou, Guangdong, China

3. Department of Mathematics and Statistics, University of Strathclyde, Glasgow, United Kingdom

4. Department of Obstetrics and Gynecology, Amsterdam University Medical Centers, location AMC, Amsterdam, The Netherlands

5. Amsterdam Reproduction and Development Research Institute, Amsterdam, The Netherlands

6. Department of Obstetrics and Gynecology, University Medical Center Groningen, University of Groningen, Groningen, The Netherlands

7. Department of Electronic and Electrical Engineering, University of Strathclyde, Glasgow, United Kingdom

8. Institute of Women and Children’s Health, School of Life Course and Population Sciences, King’s College London, London, United Kingdom

**$** Co-first authors; *****Co-last authors

**Corresponding Author**

Henk Groen,

Department of Epidemiology, University Medical Center Groningen, University of Groningen, Groningen, The Netherlands

Email: [h.groen01@umcg.nl](mailto:h.groen01@umcg.nl)

Tel: +31 6 21873277

**Online Supplement**

Table S1	Definition of outcome components and ethnicity	Page 3-4

Table S2	Sources of predictors	Page 5

Figure S1	Flowchart of participants exclusion and allocation	Page 6

Table S1. Definition of outcome components and ethnicity

| **Outcome/Variable** | **Definition** [1] |
| --- | --- |
| Ethnicity |  |
| White | Origins in Europe, Middle East, North Africa [Arabic origins], Western Russia [including Afghanistan and South Russia] and Hispanics of European origin |
| Asian | Origins in the Indian sub-continent [e.g., India, Pakistan, Bangladesh, and Sri Lanka], or in the Far East and Southeast Asia [e.g., China, Japan, Korea, Philippines, Thailand, Eastern Russia] |
| Black | Origins in any of the original peoples of Africa |
| Other | Including mixed ancestry |
| Mortality | Maternal death occurring within six weeks of pregnancy or if later, attributable to complications of preeclampsia |
| Hepatic dysfunction | International normalized ratio (INR) >1.2 in the absence if disseminated intravascular coagulation (DIC) or treatment of warfarin (DIC is defined as having both: abnormal bleeding and consumptive coagulopathy [i.e., low platelets, abnormal peripheral blood film, or one or more of the following: increased INR, increased prothrombin time (PTT), low fibrinogen, of increased fibrin degradation products that are outside normal non-pregnancy ranges]) |
| Hepatic hematoma or rupture | Blood collection under the hepatic capsule as confirmed by ultrasound or laparotomy |
| Glasgow coma score (GCS) <13 | Based on GCS scoring system: Teasdale G, Jennet B. Assessment of coma and impaired consciousness: a practical scale. *Lancet* 1974; **2**:81-83 |
| Stroke | Acute neurological event with deficits lasting longer than 48 hours |
| Cortical blindness | Loss of visual acuity in the presence of intact papillary response to light |
| Reversible Ischemic Neurologic Deficit (RIND) | Cerebral ischemia lasting longer than 24 hrs but less than 48 hours revealed through clinical examination |
| Retinal detachment | Separation of the inner layers of the retina from the underlying retinal pigment epithelium (RPE, choroid) and is diagnosed by ophthalmological exam |
| Acute renal insufficiency | For women with no underlying renal disease, defined as serum creatinine >150 μM |
| Acute renal failure | For women with an underlying history of renal disease, defined as serum creatinine >200 μM |
| Dialysis | Including haemodialysis and peritoneal dialysis |
| Postpartum hemorrhage (PPH) requiring transfusion or hysterectomy | Occurrence of PPH that required transfusion or hysterectomy |
| Placental abruption | Any occurrence of abruption diagnosed clinically or based on placental pathology report |
| Platelet count < 50,000 x 10^9^/L without blood transfusion | Measurement of platelet count recorded as less than 50,000 x 10^9^/L without patient receiving a blood transfusion |
| Transfusion of blood products | Includes transfusion of any units of blood products: fresh frozen plasma (FFP), platelets, red blood cells (RBCs), cryoprecipitate (cryo) or whole blood |
| Positive inotropic support | The use of vasopressors to maintain systolic blood pressure >90 mmHg or mean arterial pressure >70 mmHg |
| Myocardial ischemia/infarction | Electrocardiogram (ECG) changes (ST segment elevation or depression) without enzyme changes AND/OR any one of the following: 1) Development of new pathologic Q waves on serial ECGs. The patient may or may not remember previous symptoms. Biochemical markers of myocardial necrosis may have normalized, depending on the length of time that has passed since the infarct developed. 2) Pathological findings of an acute, healed or healing MI 3) Typical rise and gradual fall (troponin) or more rapid rise and fall (CK-MB) of biochemical markers of myocardial necrosis with at least one of the following: a) ischemic symptoms; b) development of pathologic Q waves on the ECG; c) ECG changes indicative of ischemia (ST segment elevation or depression); or d) coronary artery intervention (e.g., coronary angioplasty) |
| Eclampsia | Any episode of seizure antepartum, intrapartum or before postpartum discharge as follow-up beyond discharge is not possible |
| Require >50% oxygen for greater than one hour | Oxygen given at greater than 50% concentration based on local criteria for longer than 1 hour |
| Intubation other than for Caesarean section | Intubation by endotracheal tube |
| Severe breathing difficulty | Suspected pulmonary oedema where x-ray confirmation unavailable may be diagnosed by presence of chest pain or dyspnea, crackles in the lungs and SaO_2_ <90% |
| Pulmonary oedema | Clinical diagnosis with x-ray confirmation or requirement of diuretic treatment and SaO_2_ <95% |

Reference

1.	Montgomery-Csoban T, Kavanagh K, Murray P, Robertson C, Barry SJE, Vivian Ukah U, et al. Machine learning-enabled maternal risk assessment for women with pre-eclampsia (the PIERS-ML model): a modelling study. Lancet Digit Health. 2024;6(4):e238-e50. doi: 10.1016/S2589-7500(23)00267-4. PubMed PMID: 38519152; PubMed Central PMCID: PMCPMC10983826.

| Predictors from PIERS-ML model | Predictors from miniPIERS model | Candidate predictors from other literature |
| --- | --- | --- |
| **Demographic**:  Weight at eligibility (kg),  Maternal height (cm),  Maternal age at expected delivery date (years)  **Pregnancy related measurements**:  Gestational age at eligibility (weeks),  Systolic blood pressure (mmHg),  Diastolic blood pressure (mmHg),  Oxygen saturation (%),  **Proxy for quality of healthcare**:  National GDP (gross domestic product) per capita (US$),  National maternal mortality rate (per 100,000 live births) | **Symptoms**:  Headache or visual disturbances (yes/no),  chest pain or dyspnea (yes/no)  **Pregnancy related variable**:  Parity (used as ordinal variable in this study) | **Symptoms**:  right upper quadrant or epigastric pain (yes/no),  nausea or vomiting beyond mid-pregnancy (yes/no) |

**Table S2. Sources of predictors**

**Figure S1. Flowchart of participants exclusion and allocation**

(a): Model training and internal validation; (b): model external validation
